# Shock Absorbers Damping Characteristics by Lightweight Drop Hammer Test for Naval Machines

**DOI:** 10.3390/ma14040772

**Published:** 2021-02-06

**Authors:** Andrzej Grządziela, Marcin Kluczyk

**Affiliations:** Mechanical-Electrical Department, Polish Naval Academy of the Heroes of Westerplatte, 81-103 Gdynia, Poland; m.kluczyk@amw.gdynia.pl

**Keywords:** shock absorbers, UNDEX, damping, shock hammer, SRS

## Abstract

The technical requirements for naval ships machine foundations are far more strict in comparison to merchant’s vessels. These requirements are confirmed in the military standardization of many countries. Underwater Explosion (UNDEX phenomena) detonation pulses, force naval engineers to design and implement different shock absorbers made from a wide variety of materials. This study presents the tests results of typical shock absorber designs made of various types of rubber and elastomers. The initial objective of the study was to determine the energy absorption of shock impacts, the choice of materials capable of operating within the temperature range of 0 °C to 70 °C, resistance to contact with oils and marine fuel, performance at frequencies ranging from 5 to 30,000 Hz, and absorption no less than 40% of harmonic vibration energy. Initial studies conducted on tensile testing machine were used to determine the static and dynamic stiffness of a shock absorbers. Considerations of stiffness coefficient for the linear and nonlinear range is typical for shock pulses. Further tests were carried out on a lightweight drop hammer to determine the characteristics of the damping coefficient for high-speed wave interactions—Shock Response Spectrum (SRS). The final aim of the study was to assess the repeatability of the shock absorbers response to multiple impact loads. Mechanical properties describing possibilities of tested dampers materials to absorb energy of UNDEX were also presented.

## 1. Introduction

The physical effects of the underwater detonation process have been extensively studied since the mid-20th century [1]. The experience of World War II and subsequent maritime conflicts indicate that the UNDEX impulse as a noncontact explosion can significantly damage ship equipment [2]. Pulse impact, especially its first whip, can cause brittle fractures for foundations, bearings, and electronics [3,4]. Research on this phenomenon should be divided into two branches, i.e., detonation wave modelling and shock absorbers modelling for naval machines. Some publications combine both branches of study, editing recommendations and calculation procedures for naval architects.

Modelling of the detonation wave is carried out on naval training ground or in laboratories. Sea tests involve measurements and modelling of detonation wave pressure for various sea depths and seabed types. Usually, it is called Full Ship Shock Trial (FSST).

FSST tests are costly, and only a few navies can afford such experiments to be undertaken on a large scale. Tests are carried out at two-thirds of the maximum shock level in the flexible regime. Flexible regime means two concepts at the same time. The first meaning is the limited repeatability of tests on sea polygon in terms of the depth of the TNT charge location and the precise distance from the hull. The expression “flexible regime” thus means the performance of tests and measurements with the same geometrical, kinematic, and dynamic parameters as far as possible, with the assumed tolerance. The second meaning is the incomplete regime of operation of marine equipment. For security reasons, e.g., the propulsion system is in standby mode. It should be noted that even such complex component tests do not analyze the potential damage caused by the intricate coupling of several components.

Modelling and Simulation (M&S), the purpose is more often used in practice with the purpose of obtaining:

1. the assessment of the immunity of ship equipment, systems, and structures to survive UNDEX long-field shock for underwater conventional and/or nuclear detonation [5,6],
2. evaluation of existing test data and procedures to confirm validation and verification of current and anticipated new M&S capabilities.

M&S increasingly uses the Dynamic Design and Analysis Method (DDAM) in the form of numerical simulations in which three independent parts are considered, i.e., [4,7]:

- UNDEX impulse response to water, i.e., a description of the propagation of the pressure impulse in water and its impact on the immersed area of the ship.
- Structural response of the ship’s hull to the UNDEX impulse.
- The reaction of the tested component in the form of anticipated vibrations and potential damage [5].

Laboratory tests are essential during validation of the proposed simulation models. The shock wave simulator is a device for simulation and testing of one-dimensional interaction between fluid and the sandwich panel structure of the detonation test wave in test laboratory conditions [4,8,9]. The simulator is shaped like a tube filled with water in which bullets hit at one end. The test structure is placed at the opposite end of the pipe. Once the bullet hits the wall of the tube in the water, it exponentially disperses pressure impulses with peak pressures in the range of up to 70 MPa and decay times in the range of 0.1–1.5 ms are created. Peak pressure values and pulse duration are functions of the projectile speed and mass, respectively [10]. A device such as this allows a precise mathematical model of the detonation wave to be developed.

The goal of this study is to examine the reactions of the tested components to shock impulses. Literature analysis shows that many research centers place considerable attention on M&S of the machine’s foundation. The research concerns the selection of materials, construction of shock absorbers as well as configuration and foundation structure. Further branches of study include validation procedures, installations of test shock machines as well as constructional recommendations and standardization [11]. This paper presents the methodology of validation tests for small components up to 50 kg, the construction of a light shock testing machine and the results of shock absorbers tests for marine construction. The Shock Response Method (SRM) as a tool for shock absorbers evaluation is also presented.

## 2. Materials and Methods

### 2.1. Shock Machine Stands

According to [12], shock machine testing stands should be classified as specified:

- Heavyweight shock machine. The heavyweight test is a test performed on a standard or large floating shock platform without restrictions on the maximum weight of the tested device.
- Medium weight shock machine. Weight of the tested equipment including all fixings necessary to attach it to the test machine should be less than 3356.5 kg.
- Lightweight shock machine. Weight of the tested item including all fixings necessary to attach it to the test machine should be less than 249.5 kg.

#### 2.1.1. Construction of a Lightweight Shock Machine

**Figure 1:**
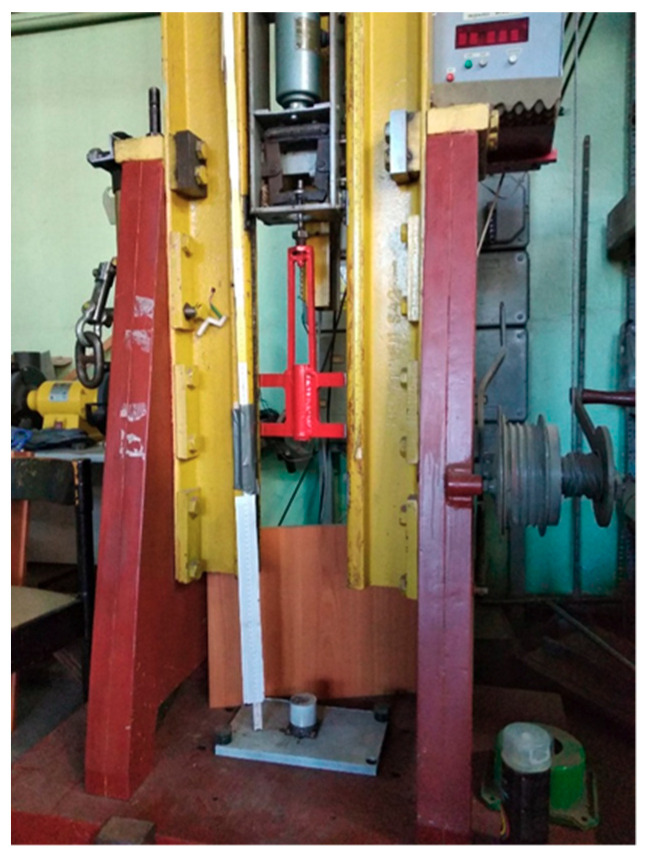
Lightweight Shock Machine in the laboratory of Polish Naval Academy.

Shock resistance tests of the shock absorbers were carried out in the laboratory of the Polish Naval Academy, using a Lightweight Shock Machine—LWSM (hammer type)—Figure 1.

The presented LWSM is used to test components weighing up to 50 kg. The machine applies a shock to the element as a result of the hammer falling from a fixed height. The test piece is founded on an anvil table, and the 5 kg drop hammer is raised to a given height. The hammer is released and converts potential energy into kinetic energy, which creates an elastic collision on the anvil table. It creates a shock pulse through the anvil table and into the component being tested. An essential element is the anvil table, whose impedance characteristics simulate the hull and ship bulkheads. The obtained signals significantly resembled a real shock of detonation [13].

#### 2.1.2. Methodology of the Shock Tests

The research methodology was divided into three stages, i.e., shock absorbers rigidity tests concerning Hook’s law, repeatability tests of results with multiple impact loads and SRS tests. The last ones were performed on LWSM in the range of up to 20% of dynamic deflection of the shock absorbers.

Tensile testing was conducted on a variety of five materials with use of hydraulic strength testing machine. The main goal was to determine properties such as ultimate tensile strength. The tensile tests involved the application of increased loads, up to 20% of compressed deformation of the length of shock absorbers.

The tests for assessing the repeatability of shock transmittance for multiple impact loads were the second step of the researches. The experiments were carried out for five types of materials. The study aimed to determine the impact of numerous shock loads on the physical properties of the absorbers, in particular, their stiffness and damping factors.

SRS tests were performed on LWSM using the Pulse system by Bruel&Kajer (Skodsborgvej, Denmark). Acceleration measurements were performed with the use of piezoelectric sensors with a sensitivity of 10 mV/g cooperating with the 6-channel input module. Maximum sampling frequency of the system is 131,072 Hz per channel. A very important feature of the input module is the spurious-free dynamic range, in this case 160 dB. Another important issue related to impulse excitation measurements is the automatic detection of sensor overloads, which allows to minimize the number of incorrect measurements. A high-pass filter was used with a cut-off frequency of 0.7 Hz and the sampling frequency was limited to 32,768 Hz. All tests were recorded using a slow-motion camera that enables image recording with a resolution of 960 frames per second

It was assumed simplification that the falling hammer with shock absorber constitute a homogeneous mass and might be assumed as a single degree of freedom (SDOF) system.

### 2.2. Tensile Testing of the Shock Absorbers

**Figure 2:**
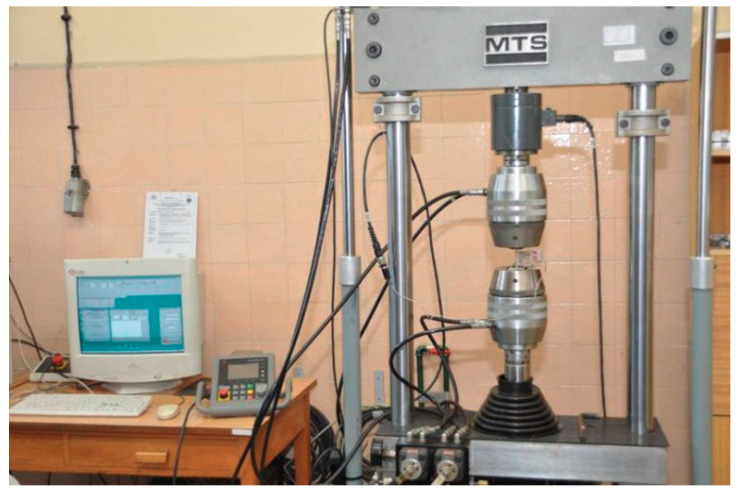
Measure, test, simulate (MTS) tensile testing machine.

Tensile testing was conducted using the machine in the Laboratory of Technics Principles at the Polish Naval Academy—Figure 2.

**Figure 3:**
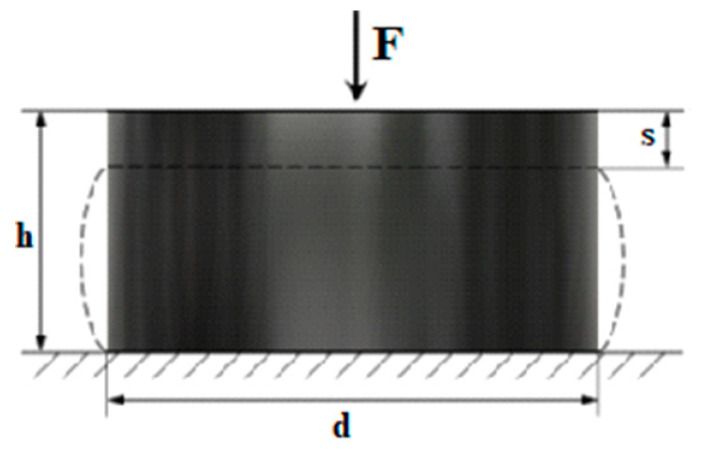
Scheme of tensile tests of shock absorbers.

Hydraulic strength testing machine allowed for accurate and repeatable tests on five types of shock absorbers. Digital controllers provided fast closed-loop control and a 100 Hz data acquisition rate. Load cells were checked during testing to ensure high rigidity and stability with low nonlinearity. The research assumed that the maximum deflection would not be more than 20% of the height of the shock absorber—Figure 3:

(1) $$\frac{s}{h}<0.2\;h.$$

In fact, rubber is a nonlinear material, but in the applications of naval machine foundation, the shock absorbers or dampers deformation coming from weight, static displacement, and harmonic oscillation as well as far field underwater explosions should be not greater than 20%. For such a deformation, the elasticity is slightly nonlinear, so the authors adopted a linear model for theoretical considerations.

During the various tests carried out on the shock absorbers five types of materials, each with three most popular in marine industry levels of hardness, i.e., 55°, 65°, and 75° Sh A (according to [14]) were tested. A nitryl rubber (NBR) is the most popular material for shock absorbers in marine power plants applications and four additional materials were added as a possible alternative. The following materials were applied in the production of shock absorbers:

- CR—rubber based on chloroprene rubber,
- EPDM (ethylene propylene diene monomer)—rubber cross-linked in the process of sulfur vulcanization,
- NBR (nitrile rubber)—commonly known as oil-resistant rubber, i.e., a copolymer of butadiene and acrylonitrile,
- NR—natural rubber, also called rubber, is a flexible hydrocarbon polymer,
- PU—polyurethane is a polymer, which contains the urethane group NH-CO-O formed by combining hard (isocyanate) and elastic (polyol) parts.

### 2.3. Hammer Testing of Shock Absorbers

**Figure 4:**
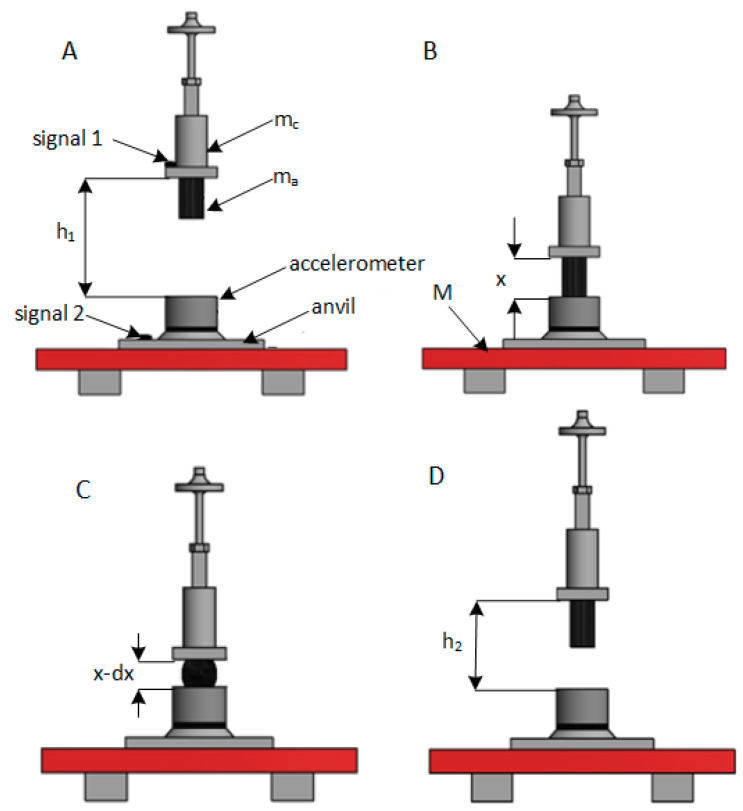
Diagrams of theoretical foundations for conducted research. (**A**) initial hammer drop position, (**B**) point of absorber contact with the anvil, (**C**) point of maximum absorber deflection, (**D**) point of maximum rebound height.

The hysteresis effect for dampers and shock absorbers is well known and used in the design of devices operating under impact loads (see Figure 4).

The tests on LWSM were carried out according to the following theoretical assumptions. The bouncing of shock absorbers from the anvil includes initial gravitational potential energy $E_{P1}$, kinetic falling and rebound energies $E_{K1}$ and $E_{K2}$, and elastic potential energy $E_{PL}$. When the hammer with the shock absorber falls, its gravitational energy $E_{P1}$ transforms into $E_{K1}$. Because the height is relatively small, the resistance of air friction might be neglected. When the hammer made contact with the anvil, its $E_{K1}$ was equal to $E_{P1}$, which it already had initially. After the moment of contact, the shock absorber compressed, and the kinetic energy was stored as elastic $E_{PL}$ and dissipated by damping energy $E_{D1}$ during the compression of the absorber (mainly by heating). When the sum of $E_{PL}+E_{D1}$ reached the value of $E_{K1}$, the compressed absorber stopped, and the entire $E_{PL}$ turned into $E_{K2}$. However, during expansion, a part of $E_{PL}$ was consumed by $E_{D1}$. Then, the hammer rose to a new, lower height at which it obtained $E_{P2}$ potential energy. The work assumes that during deformation, elastic energy is stored in the shock absorber, which is released after removing the deformation forces. Shock absorbers are made of viscoelastic materials; hence why some of its energy is dissipated in the form of heat. The energy consumed in the tests was considered as damping energy ($E_{D}=E_{D1}+E_{D2}$).

The shock absorber was mounted in LWSM at a height of $h_{1}$, then dropped. Next, the shock absorber bounced off the anvil, and the results of its deflection ∆*x* were recorded using a fast camera that allowed videos to be played in slow motion. The following assumptions were made during the tests:

The mass *M* of the LWSM foundation is far greater than the mass *m*, where

(2) $$m=m_{c}+m_{a}$$

$m_{c}$—the mass of the hammer,

$m_{a}$—the mass of the shock absorber,

$h_{1}$—height from the ground to the center of gravity of the hammer and shock absorber *m*, i.e., falling displacement $x_{1}$.

A drop hammer with installed shock absorber (total mass of system is *m*) at height $h_{1}$ possesed potential energy equal to

(3) $$E_{P1}=mgh_{1}.$$

Additionally, the next derivatives of the displacement had the following initial conditions

$$\dot{x_{1}}\left(t=0\right)=0\;\;\;\;\;\;\;\;\;\;\;\;\;\;\;\;\;\;\;\;\;\;\;\;\;\;\;\;\;\;\;\;\;\;\;x_{1}\left(t=0\right)=\;h_{1}\;\;\;\;\;\;\;\;\;\;\;\;\;\;\;\;\;\;\;\;\;\;\;\;\;\;\ddot{x_{1}}\left(t=0\right)=g.$$

Then the hammer falling from a height of *h*_1_ hits the foundation with a mass *M*, where its potential energy $E_{P1}$ is converted into kinetic energy *E_K_*_1_ for the final speed of movement.

(4) $$E_{P1}=E_{K1},$$

where

(5) $$E_{K1}=m\frac{v^{2}}{2}=\frac{m}{2}{\left(\dot{x}\right)}^{2}\;\;\;for\;\;\;h=0.$$

Simplifications in the physical model were adopted:

- no air resistance,
- the shock absorber base is in parallel contact with the *M* foundation,
- the acceleration of the mass of the anvil is neglected due to its significantly greater mass
- anvil is completely rigid bod.

Thus, the speed of the drop hammer together with the shock absorber was determined as follows

(6) $$mgh_{1}=\;m\frac{v^{2}}{2}$$

(7) $$v=\;\sqrt{2gh_{1}}.$$

At the moment of impact, the shock absorber on the anvil deflects with the movement of the center of gravity of mass as a result of kinetic energy *E_K_*_1_ conversion into the sum of elastic energy *E_E_*_1_ and damping energy *E_D_*_1_.

(8) $$E_{K1}=E_{E1}+E_{D1}.$$

This conclusion allows for determination of the damping coefficient c later on in the study. Simplifications of the physical model have been adopted for preliminary tests at low altitudes:

- *k*—the elasticity of the shock absorber is assumed to be linear (Hooke’s law for small drop height in which deflection of absorbers do not exceed 20% of their initial height)
- *c*—suppression as dissipation of all forms of energy.

Thus, equation of balanced forces can be written as follows

(9) $$m\Delta \ddot{x}=k\Delta x+c\left(\Delta \dot{x}\right).$$

In addition, equation of balanced energies is

(10) $$m\frac{v^{2}}{2}=k{\left(\Delta x\right)}^{2}+c\left(\Delta x\right)\left(\Delta \dot{x}\right).$$

The center of gravity displacement is equal to *x* because in the hammer position C (see Figure 4, the kinetic energy was converted into elastic energy and dissipated by damping. It was assumed a simplification that the damping energies during compression and extension of the shock absorber are equal, so the velocity of the movement can be written as

(11) $$v=\sqrt{2gh_{1}}=\left(\Delta \dot{x}\right),$$

where

kΔx—spring force of absorber,

$c\Delta \dot{x}$—damping force, where *c* is damping coefficient.

Since the hammer speed was measured by the optical method, Equation (11) was to verify the theoretical assumptions. The error did not exceed 2%. Position D of the hammer (see Figure 4 is preceded by moment when *E_E_*_2_ is converted into the kinetic energy caused by the upward movement of the hammer—*E_K_*_2_ and then the kinetic energy is transformed into potential energy at the final height *h*_2_ of *E_P_*_2_. The initial velocity of the shock absorber in its reflected position from the anvil is equal to *v*_2_.

According to (9), the damping coefficient might be calculated as

(12) $$c=\;\frac{m\left(\Delta \ddot{x}\right)-k\Delta x}{\Delta \dot{x}}.$$

Because the hammer, due to the elastic nature of the shock absorber, is rejected to a height of $h_{2}$, all elastic energy $E_{E1}$ is converted into potential energy $E_{P2}$

(13) $$E_{E1}=k\Delta x^{2},$$

where

(14) $$E_{P2}=mgh_{2}.$$

Thus, the modulus of elasticity *k* of the shock absorber can be determined as

(15) $$k\Delta x^{2}=mgh_{2}$$

(16) $$k=\frac{1}{\Delta x^{2}}mgh_{2}.$$

### 2.4. Shock Response Spectrum as a Tool of Damping Determination

**Figure 5:**
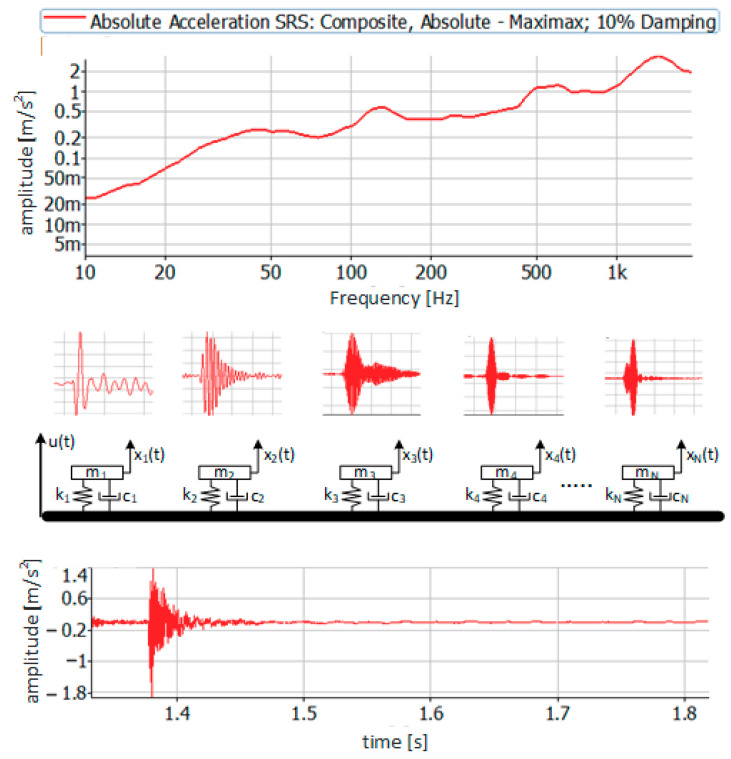
Graphical interpretation of Shock Response Spectrum (SRS) creation.

Shock Response Spectrum (SRS) is created as the sum of the responses of single systems with one degree of freedom (SDOF), all of which are subjected to the same impulse excitation [2,15,16,17]. Therefore, it is a system response presented as individual maximum accelerations (positive or negative). It should be emphasized that subsequent systems with one degree of freedom are characterized by increasing stiffness and, consequently, natural frequencies. Therefore, in the high-frequency band, the SRS spectrum obtains amplitudes close to the value of the excitation signal amplitude (transmittance close to unity) [18]. A graphic illustration of the principle of SRS spectrum creation is shown in Figure 5.

Assuming that each of the oscillators has an independent absolute displacement coordinate $x_{n}\left(t\right)$, and the coordinate of the displacement of the foundation is u(t) (assuming only one direction of movement), in the case of impact on the acceleration foundation $\ddot{u}\left(t\right)$ each of the oscillators will respond in a characteristic way [19]. The equation of motion of the *n*-th oscillator can be written as

(17) $$m_{n}{\ddot{x}}_{n}\left(t\right)+c_{n}\left({\dot{x}}_{n}\left(t\right)-\dot{u}\left(t\right)\right)+k_{n}\left(x_{n}\left(t\right)-u\left(t\right)\right)=0$$

mass displacement relative to the base $z_{n}$ is described by

(18) $$z_{n}\left(t\right)=x_{n}\left(t\right)-u\left(t\right).$$

After substitution

(19) $$m_{n}\left({\ddot{z}}_{n}\left(t\right)+\ddot{u}\left(t\right)\right)+c_{n}{\dot{z}}_{n}\left(t\right)+k_{n}z_{n}\left(t\right)=0.$$

After transformations, it might be written as

(20) $${\ddot{z}}_{n}\left(t\right)+\frac{c_{n}}{m_{n}}{\dot{z}}_{n}\left(t\right)+\frac{k_{n}}{m_{n}}z_{n}\left(t\right)=-\ddot{u}\left(t\right).$$

Remembering that $\frac{k_{n}}{m_{n}}=\omega_{n}^{2}\;$($\omega_{n}$—the natural angular frequency of the *n*-th oscillator) and $\frac{c_{n}}{m_{n}}=2\zeta_{n}\omega_{n}$, ($\zeta_{n}$—the percent of critical damping), Equation (17) might be written in the following form

(21) $${\ddot{z}}_{n}\left(t\right)+2\zeta_{n}\omega_{n}{\dot{z}}_{n}\left(t\right)+\omega_{n}^{2}z_{n}\left(t\right)=-\ddot{u}\left(t\right).$$

Using the previous Equations (17)–(20), the acceleration of mass $m_{n}$ can be determined as follows

(22) $${\ddot{x}}_{n}\left(t\right)=-2\zeta_{n}\omega_{n}{\dot{z}}_{n}\left(t\right)-\omega_{n}^{2}z_{n}\left(t\right).$$

Assuming the damping in SRS is negligible, Equation (22) could be written in the following form

(23) $${\ddot{x}}_{n}\left(t\right)≅-\omega_{n}^{2}z_{n}\left(t\right).$$

It can be stated that the acceleration of the *n*-th mass is proportional to the relative displacement of the mass considered and its foundation. Equation (21) can be solved using the Duhamel integral

(24) $$z_{n}\left(t\right)=-\frac{1}{\omega_{n}\sqrt{1-\zeta^{2}}}\int_{0}^{t}\ddot{u}\left(\tau \right)e^{-\zeta_{n}\omega_{n}\left(t-\tau \right)}sin\omega_{n}\sqrt{1-\zeta^{2}}\left(t-\tau \right)d\tau .$$

After substitution of (24) to (23), it will be

(25) $${\ddot{x}}_{n}\left(t\right)=-\frac{\omega_{n}}{\sqrt{1-\zeta^{2}}}\int_{0}^{t}\ddot{u}\left(\tau \right)e^{-\zeta_{n}\omega_{n}\left(t-\tau \right)}sin\omega_{n}\sqrt{1-\zeta^{2}}\left(t-\tau \right)d\tau .$$

Remembering that SRS is the absolute value of vibration accelerations for individual frequencies $\omega_{n}$, for *n* frequencies, it can be written as follows

(26) $$SRS={\left|{\ddot{x}}_{n}\left(t\right)\right|}_{max}.$$

Finally, the equation for the system SRS consisting of *n* oscillators with one degree of freedom (SDOF) might be written as

(27) $$SRS_{n}={\left|\frac{\omega_{n}}{\sqrt{1-\zeta^{2}}}\int_{0}^{t}\ddot{u}\left(\tau \right)e^{-\zeta_{n}\omega_{n}\left(t-\tau \right)}sin\omega_{n}\sqrt{1-\zeta^{2}}\left(t-\tau \right)d\tau \right|}_{max}.$$

Remembering that

(28) $$f_{n}=\frac{\omega_{n}}{2\pi },$$

SRS as a frequency function might be obtained.

## 3. Results

This section presents results of researches conducted with methods described in the previous chapter. Each subsection corresponds to one described method.

### 3.1. Tensile Testing of Shock Absorbers

**Figure 6:**
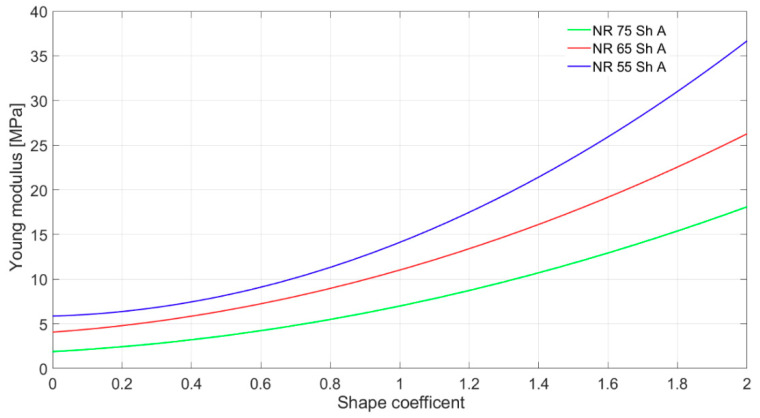
The results of the static measurements of shock absorbers stiffness made of three natural rubber (NR) rubber types.

Stress relaxation occurs in loaded elastomers, i.e., the stress value at a given elongation decreases over time. Therefore, with slow stretching, lower values of corresponding stresses are obtained than with faster ones [7,20,21]. When assessing the mechanical properties, it is necessary to provide the stretching or compressing speed. Mullin and Payne dealt with these phenomena [22]. The research draws comparisons between the shock absorbers stiffness value for static and for high-velocity deflection. The deformation velocity is to be in the same order of magnitude of the deformation velocity as the UNDEX effect [1]. An example of the result of static measurements of shock absorbers stiffness made of rubber NR type is shown in Figure 6. A shape coefficient is described by Equation (29)

(29) $$w=\frac{d}{4h},$$

where *d* is largest absorber rubber part diameter at selected load (see Figure 3) and *h* is absorber height at selected load.

**Figure 7:**
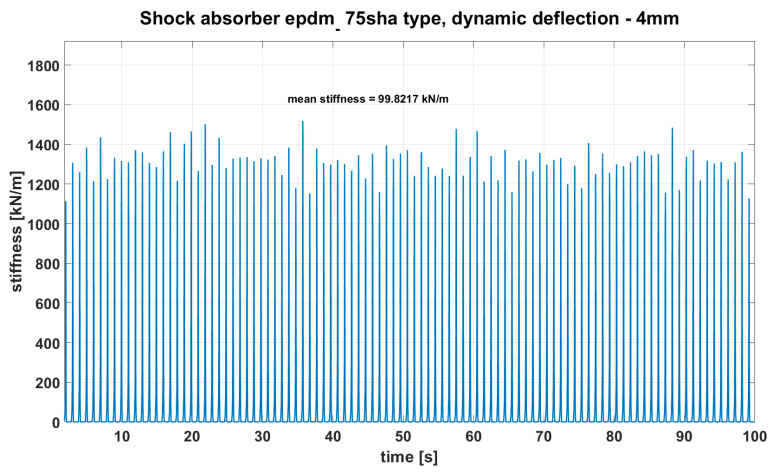
Result of dynamic measurements of shock absorber’s stiffness made with velocity 5 m/s and probe frequency 0.9 Hz, material 75 Sh A EPDM.

Sample stiffness change test for 20% deflection and velocity *v* = 5 m/s on shock absorber EPDM 75 Sh A type (tests were conducted on a tensile machine presented on Figure 2) is shown in Figure 7.

An analysis of the test results for all types indicates that with increasing speed, the stiffness for all kinds of shock absorbers increases. During the tests, it was noticed that the stiffness values for the set strain rate were not constant and that they changed significantly—see Figure 7. The average stiffness values are presented in Table 1.

### 3.2. Hammer Testing of Shock Absorbers

**Figure 8:**
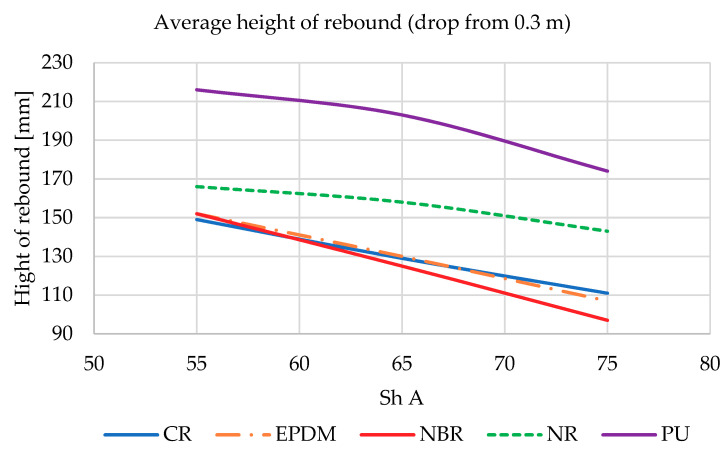
Hight of rebound of all tested shock absorbers dropped from 0.3 m.

**Figure 9:**
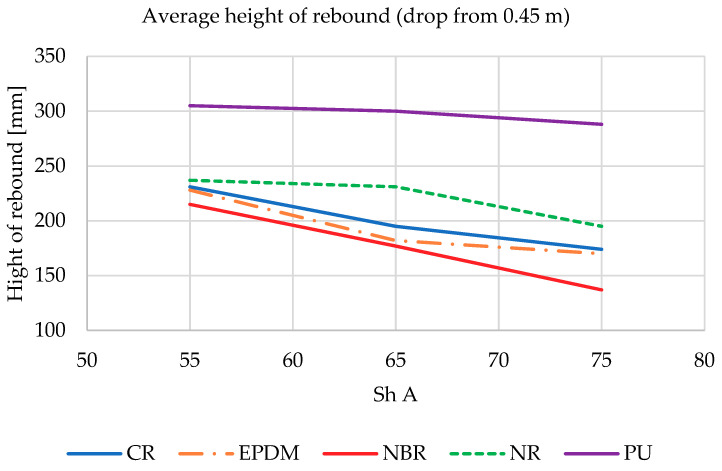
Hight of rebound of all tested shock absorbers dropped from 0.45 m.

The *k* coefficient described by Equation (16) from the drop hammer experiment is proportional to the heigh of rebound. During testing on LWSM, a fast camera with automatic object detection in the frame and an image recording speed of 960 fps were used to record the deflection distance and height of the shock absorbers bounce. Recordings were processed caged to obtain the images of the compressed shock absorbers and the maximum height of rebound. At the same distance from the camera lens as the falling shock absorber, a prepared vertical ruler was placed, which was used as a distance reference during further processing. Henceforth, during the caged image processing, it was also possible to determine the contact time of the shock absorber with the anvil of the LWSM, which was used in the latter part of the analysis. Results of the test are presented on Figure 8 and Figure 9.

It was noticed that in all materials used in the shock absorbers, the reflection height decreased as the hardness (Sh A) increased. Described situation occurs for both heights of drop (0.3 m—Figure 8 and 0.45 m—Figure 9). This clearly shows that the dissipation of the impact energy in the case of lower material stiffness is lower (stiffness as it was presented in Table 1 is connected with the hardness of tested materials). The best result was obtained by an absorber made of NBR rubber with a hardness of 75 Sh A.

### 3.3. Shock Response Spectrum as a Tool of Damping Determination

**Figure 10:**
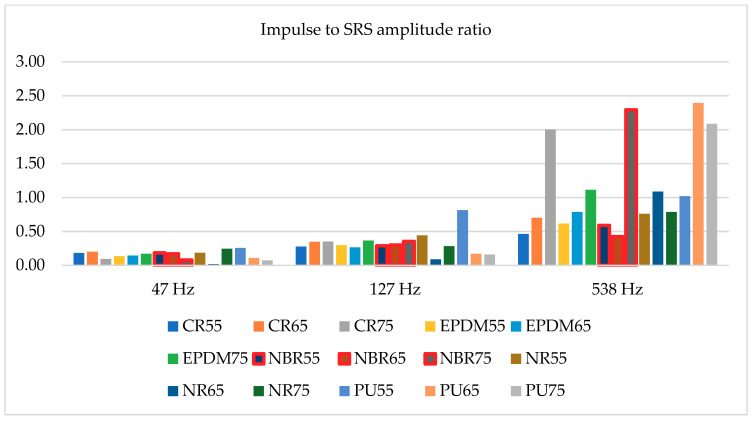
Impulse to SRS amplitude ratio for hammer drop down from 300 mm.

**Figure 11:**
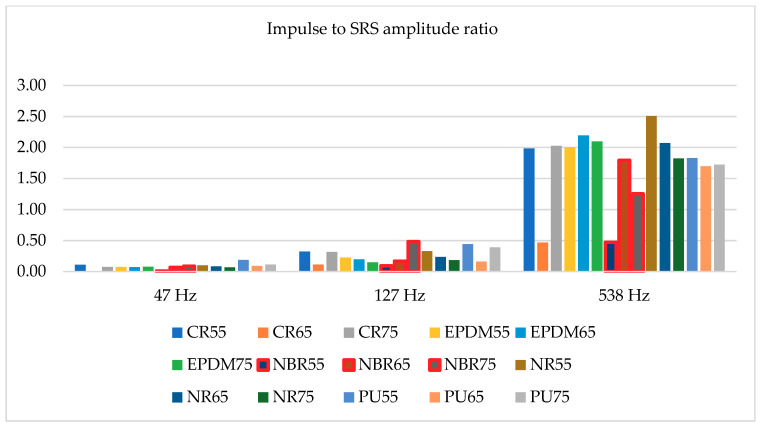
Impulse to SRS amplitude ratio for hammer drop down from 450 mm.

Fifteen different shock absorbers were tested, all of them having the same geometrical dimensions, i.e., cylinder diameter 20 mm with a height of 40 mm. Among them five different materials were tested (described above in the paper), each of them with three different hardness (55, 65, and 75 Sh A). All tested absorbers were dropped down from 300 and 450 mm. Due to the fact that the impulse of acceleration recorded on the hammer drop test bed was not always duplicated, selected SRS amplitudes were divided by maximum acceleration during each test. As a result, two charts were created—Figure 10 and Figure 11. Most important, three different SRS amplitudes were taken into consideration with frequencies: 47, 127, and 538 Hz.

Figure 10 relates to impact tests conducted from a height of 0.35 m, and Figure 11 from a height of 0.45 m. The accelerations at the moment of contact of the shock absorbers with the ground were different in relation to both heights, but the presented results are similar. This is due to the fact that the maximum values of the considered SRS amplitudes are compared to the maximum values of the accelerations recorded during individual impacts. The values presented in Figure 10 and Figure 11 state that the ratios of the impulse acceleration amplitudes to the amplitudes of the SRS accelerations do not depend on the hardness of individual materials (Sh A). This is due to the different dynamic stiffness of individual materials used for the production of metal-rubber shock absorbers [23]. Usually, the manufacturers of these elements do not give the value of dynamic stiffness because it is not important in “civil” applications. By analyzing data presented in Table 1, it may be stated that there is a nonlinear dependence between shock absorbers hardness and the dynamic stiffness. However, considering the situation in which the metal-rubber element, in addition to minimizing the value of vibration parameters related to the operation of the machine, is also used to protect this machine from the effects of an underwater detonation wave (like all devices mounted on navy ships), its rigidity at high deformation speeds turns out to be extremely important (the shock wave in water propagates at a speed of about 1500 m/s) [5]. According to this knowledge the values presented in Figure 10 and Figure 11 might be reanalyzed. Consequently, the best material with regards to protection of the devices against impulse effects among the presented materials is NBR rubber (values surrounded by red lines), specifically with a hardness of 55 Sh A. According to results of static stiffness, it might be stated that the ability to dissipate energy connected with underwater explosion impact should not be strictly connected with these parameter (not all shock absorbers made of materials with the lowest stiffness tested responses with the highest energy dissipation). Therefore, the use of SRS analysis allows for a preliminary estimation of rubber materials to impulse loads, without the need to determine their dynamic and static stiffness characteristics.

## 4. Discussion

The production of vibroisolators is common and mainly aimed at civilian customers. The basic features are good harmonic vibration damping, durability, and resistance to the environment [24]. The military use of shock absorbers imposes additional technical requirements related to the damping of impact loads. Unfortunately, often the selection of dampers in terms of the correct damping of harmonic vibrations is not synonymous with the high damping coefficient of impulse impacts, the source of which in the case of navy ships is usually underwater explosions.

Five different materials used for shock absorbers production were tested (CR, EPDM, NBR, and NR rubber as well as a polyurethane), all of them with three different hardness. Investigated absorbers were tested on a tensile machine and during hammer testing on lightweight shock machine. The conducted tests confirmed the necessity of individual verification procedures for materials used for military shock absorbers series. The tests carried out indicate that the most important factor is the dynamic stiffness, that determines the dissipated elastic energy. Its high values protect machine against too big displacements. Excessive displacement of the device’s foundation due to a low coefficient of dynamic stiffness can cause failure, especially if rotating machines are exposed to it. On the other hand, many electronic devices require acceleration reduction due to the possibility of brittle fracture. Proper selection of shock absorbers for military applications is, therefore, the result of optimization of the predicted loads and permissible displacements and accelerations. Material researches performed by tests are not so expensive and, therefore, seems like a real solution to a design task. Further research conducted by the authors consists of the rheological analysis of the influence of harmonic vibrations on changes in stiffness and damping coefficients in materials used for shock absorbers.

The obtained results refer to the measurement results for the quasi-linear response of the absorbers. The conditions of real deformation of absorbers often exceed 20% deformation, hence, this is the motivation for the authors to continue research in the field of system response for nonlinear deformations (more than 20%) and to analyze rheological wear resulting from the action of harmonic forces and pulses shock types.

Tests aimed for proper selection of shock absorbers used with marine equipment should include both LWSM and SRS tests. This is due to the fact that an important feature of shock absorbers is the ability to absorb kinetic energy and convert it into damping energy. The results of the SRS tests indicate the quantitative nature of the damping by indicating the acceleration values resulting from the UNDEX impact. Shock absorbers research should be carried out in terms of selection for specific devices and not as catalogue proposals.

## Figures and Tables

**Table 1:** Averaged stiffness values of shock absorber for 4 mm and 5 m/s dynamic deflection.

Material and Its Stiffness Sh A	Deflection	Stiffness (kN/m)	High Speed Stiffness (kN/m)
CR55	4 mm	41.7	59.2
CR65	4 mm	69.2	95.3
CR75	4 mm	121.8	159.8
EPDM55	4 mm	50.7	72.1
EPDM65	4 mm	54.3	79
EPDM75	4 mm	69.1	99.8
NBR55	4 mm	44.2	54.1
NBR65	4 mm	53.8	72.2
NBR75	4 mm	105.6	141.9
NR55	4 mm	53.4	76.4
NR65	4 mm	57.3	81.4
NR75	4 mm	94.5	127.2
PU55	4 mm	44.8	64.2
PU65	4 mm	68.4	89.1
PU75	4 mm	117.4	150.1

## Data Availability

The data presented in this study are available on request from the corresponding author. The data are not publicly available due to the fact that they were obtained during research grant which is not finished yet.
